# Investigating the Impact of the Degree of Sharpness on the Microstructure of Fresh-Cut Apples

**DOI:** 10.3390/foods14040636

**Published:** 2025-02-14

**Authors:** Alessia Incardona, Maria Luisa Amodio, Antonio Derossi, Giancarlo Colelli

**Affiliations:** Department of Agriculture, Food, Natural Resources and Engineering (DAFNE), University of Foggia, Via Napoli 25, 71122 Foggia, Italy; alessia.incardona@unifg.it (A.I.); marialuisa.amodio@unifg.it (M.L.A.); antonio.derossi@unifg.it (A.D.)

**Keywords:** cutting, degrees of sharpness, X-ray micro-CT, quality, color, browning

## Abstract

Mechanical damage significantly affects the quality and shelf-life of fresh-cut produce. Understanding and controlling the effects of mechanical damage is essential for developing nutritionally rich and sensorially acceptable products. This study investigated how the efficacy of cutting tools may cause mechanical damage to the tissues of Golden Delicious apples, affecting their main physical, chemical, and microstructural properties. The apples were sliced using one kitchen knife with four Degrees of Sharpness (DoS), from sharp (DoS1) to blunt (DoS4). Over 15 days of storage, the apples cut with a DoS1 maintained a higher L* value of 80.1, with minimal changes in the a* value. The apples cut with a blunt knife (DoS4) showed a significant decrease in L* to 78.1 and an increase in the a* value from 1.2 to 3.3. X-ray micro-CT imaging revealed that the porosity at the surface of the apples cut with a DoS1 was 15%, compared to 19% for those cut with a DoS4. Additionally, the DoS4 samples showed greater structural separation at the cut surface, suggesting a larger solid fraction and lower overall quality. This study concludes that the use of blunt tools can cause damage that negatively impacts the overall post-cut quality as a result of the changes induced in the internal microstructure.

## 1. Introduction

The shelf-life period of fresh-cut fruits and vegetables (FVs) significantly relies upon the optimal unit operations involved in the fresh-cut industry [1]. Fresh-cut produce is prone to mechanical damage during the harvesting, processing, packaging, transportation, and storage phases. Operations such as cutting, peeling, slicing, and shredding induce mechanical damage resulting in tissue stress, and cytoplasm exposure resulting from the surface cell destruction, which provides a favorable environment for microorganisms and ultimately impacts the overall quality as well as consumer acceptability and profit enhancement [2]. Due to the increasing legislative and consumer demands for healthy, nutritious, ready-to-eat, and safe FVs, the food industry is focusing on the development of innovative solutions for the quality maintenance of fresh-cut produce.

Cutting is one of the major operations in the preparation of FVs. As a result of cutting, mechanical damage occurs which compromises the quality of the fresh-cut products. Biochemical, physiological, and morphological changes occur on the cutting surface resulting in a reduced shelf-life as well as lower aesthetic appeal for consumers [3]. In terms of biochemical responses, molecules such as reactive oxygen species (ROS), 1-aminocyclopropane-1-carboxylic acid (ACC), and metabolites such as phenolic compounds are released [4,5]. As an effect of the activation of biochemical responses, the wounded surface produces ethylene together with a boost in the respiration rate [6], until the appearance of morphological responses including browning, translucency, and a loss of firmness occur.

The sharpness of the cutting blade plays a significant role in the amount of mechanical damage inflicted and, in turn, on the quality attributes of fresh-cut FVs [7]. Melon cylinders cut with a blunt blade exhibit increased ethanol concentrations, off-odor scores, and electrolyte leakage, and have a higher potential for ethylene production in comparison to pieces processed with a sharp blade [8]. The utilization of sharp cutting tools may reduce wound response, lignin accumulation, white blush, softening, and microbial growth in fresh-cut carrots [9]. In fresh-cut eggplants, the use of a sharp blade followed by dipping in water can strongly reduce browning [10]. Minimal color changes and chemical responses on the cut surface can be detected when fresh-cut apples are processed with blades with a high degree of sharpness [11].

The geometrical parameters of a blade, such as wedge angles and tip radii, have been shown to hold great significance regarding blade cutting efficiency and the extent of the damage inflicted [12,13]. Moreover, the correlation between the edge angle and the other components involved in the cutting procedure, such as the speed of the cut, also needs to be considered. As the blade sharpness, cutting speed, and cutting angle play a significant role in the extent of mechanical damage, it is imperative to use micro-structural data analysis approaches to determine the extent of the damage on the cut surface. In the modern food industry, probing the microstructure of food samples has become very useful since it allows for the use of a rationale design of the processes for quality enhancement. Among the techniques used to explore the microstructures of foods, X-ray micro-computed tomography (micro-CT) generates stacks of high-resolution images which can be transformed into digital three-dimensional (3D) models of the samples under consideration. The internal structures of biological samples are investigated based on the differences in the strength of X-ray attenuation. This technique is superior to other microstructural investigating methods, such as light or electron microscopy, due to many reasons: firstly, the samples can be investigated in their natural state at the appropriate atmospheric pressure and temperature; secondly, it possesses a much higher spatial resolution as compared to the other methods; and, thirdly, X-rays possess the potentiality of (non-destructively) penetrating through the samples while capturing their 3D details, while other techniques require an invasive analysis [14,15,16,17].

Various research works proposed the use of X-ray micro-CT for the microstructural analysis of food samples such as dried fruits [18], distillers’ spent grain pellets [19], starch gluten matrices [20], Japanese apricots and pears [21], apples, turnips, eggplants [22], and bread [23]. Prawiranto et al. [18] investigated the porosity and pore diameter of apple tissues under three different drying conditions. The study concluded that a high degree of cell deformation and a higher pore size were observed in forced convective dried apples. Erkinbaev et al. [19] used the micro-CT technique to unravel the changes in distillers’ spent grain (DSG) pellet pore structures as a result of drying. Karmoker et al. [21] determined the porosity of Japanese apricots and pears stored at two different temperatures using X-ray micro-CT. It was observed that for the samples stored at 25 °C porosity increased, and the study concluded that the internal structure of a pear was well preserved at 5 °C. These results were obtained by visualizing the porosity distributions using micro-CT, proving that micro-CT is a reliable, non-destructive method of assessing and monitoring the microstructural changes in fruit samples. On the other hand, Nugraha et al. [22] investigated the potentiality of micro-CT for the porosity mapping of fruits and vegetables during postharvest storage. The study found a significantly high correlation between the porosity and greyscale values, resulting in the formulation of a 3D porosity distribution map for apples, pears, and eggplants using a single correlation factor. Clear porosity differences were also found among the various tissues, vascular bundles, and seeds present in the samples. The eggplants possessed the most porous structure followed by the turnips, apples, and pears. Conclusively, the heterogeneity of the tissue microstructures was clearly observed by the porosity maps generated using X-ray micro-CT.

The microstructures of whole apples have been extensively studied, with X-ray micro-CT demonstrating variations in physiological and morphological responses across the different vegetative tissues [24,25,26,27].

Nevertheless, to the best of our knowledge, none of the studies have yet explored the changes in microstructural properties of biological samples exposed to mechanical damage. Mechanical damage results in a higher respiration rate, affecting the transfer phenomenon which is affected by porosity. Therefore, the aim of this study is to investigate the potentiality of X-ray micro-CT for determining the changes in the microstructural profile of apple samples using four cutting conditions.

## 2. Materials and Methods

### 2.1. Sample Preparation

“Golden Delicious” apples (*Malus domestica*) were purchased from a fresh local market (SSC 12.85 ± 0.52, TA as a percentage of malic acid 0.54 ± 0.03) and processed in the postharvest laboratory of the University of Foggia. Thirty-two apples were divided into four sets and each of these was cut with one kitchen knife, each of a different Degree of Sharpness (DoS). Four DoS values, defined as the cutting force exerted to cut a reference body [12], for the knives were used to cut the apples. These values, labeled as DoS1, DoS2, DoS3, and DoS4 and corresponding to 30, 100, 140, and 190 N (Newton), respectively, were obtained by de-sharpening the knives with sand paper [11]. The eight apples of each group were cut lengthwise, then in halves along the z axis, obtaining 71, 80, 70, and 68 slices for DoS1, DoS2, DoS3, and DoS4, respectively. For the initial physical assessments, 17 slices of each treatment were used, while the remaining were stored in a perforated clamshell in a cold room at 5 °C for a storage period of 15 days, making sure that the paper tissue in each clamshell was always kept wet in order to limit the water loss from transpiration from the slices. A quality evaluation was performed after 5, 12, and 15 days of storage.

### 2.2. Color Measurement

The color parameters of the fresh-cut apples were measured using a spectrophotometer (Konica Minolta CM 2600d, Tokyo, Japan). The measurements were acquired from each slice on both sides (with the number of slices varying from 17 to 20 for each treatment) on every sampling day and changes in color were defined in the CIE L*a*b* color space [28].

Different indexes, e.g., Browning Index (BI) [11], were calculated from L*, a*, and b* values as follows:

Hue Angle h° = arctg (b*/a*)(1)

Chroma = √(〖a*〗^2^ + 〖b*〗^2^) (2)

∆e = [(ΔL*)^2^ + (Δa*)^2^ + (Δb*)^2^]^(1/2)^ (3)

Browning Index (BI) = (x − 0.31)/0.17 × 100 with x = (a* + 1.75L*)/(5.645L* + a* − 3.012 b*) (4)

### 2.3. Visual Appearance Score

The visual acceptability score, based on the RGB images taken during storage, uses a 5-point scale [29]: 5 means just cut, 4 is very good, 3 marks the limit of marketability, 2 is the limit of usability, and 1 denotes inedibility.

### 2.4. Microstructural Analysis

X-ray micro-computed tomography (micro-CT) of the fresh-cut apples was performed using a SkyScan 1174 micro-CT scanner (Brüker, Kontich, Belgium) on parallelepiped-shaped samples of 3 mm in length, 3 mm in width, and 2 cm in height, from the slices cut with a DoS1 and DoS4, at a pixel resolution of 6.5 µm. Scanning was performed on three samples taken from different slices for each considered DoS value. Nrecon 1.6.2.0 software (Brüker, Kontich, Belgium) was used to perform image processing. The entire stack of the images was cropped to obtain a region of interest, ROI, of 400 × 400 pixels corresponding to 2.6 mm^2^ of the sample representing 80% of the surface of the sample. A segmentation algorithm was used to obtain a binarized image where the white pixels represent the void phase while the black pixels represent the solid phase. The main morphological features of the pores and solid phase were measured by using the software CTAn 1.12.00 (Bruker, Belgium). In particular, the porosity fraction (%) was computed with the ratio of the total number of black pixels included in the ROI to the total number of pixels of the ROI; in addition, structure separation and structure thickness were measured by using the CTAn manual for morphometric parameters [30].

### 2.5. Statistical Analysis

For data related to color measurement and to visual appearance score, Stat Graphics Centurion XVI.I (Stat Point Technologies, Inc., Warrenton, VA, USA) was used as statistic software to perform the analysis of variance with a two-way ANOVA for the parameters assessed. Mean values of all color readings were separated with Tukey’s test at *p* ≤ 0.05.

## 3. Results and Discussion

The results of the statistical analysis of the physical parameters are presented in Table 1.

**Figure 1:**
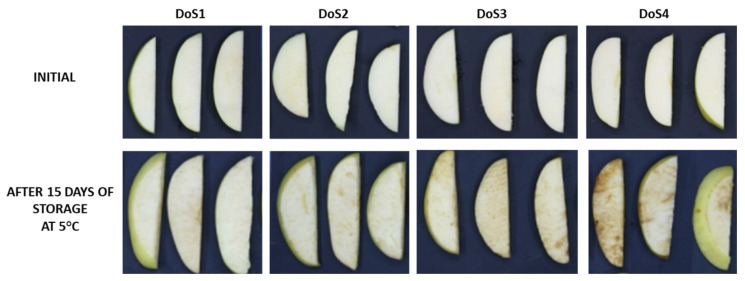
RGB images of fresh-cut apples treated with four different DoS initially and after 15 days of storage at 5 °C.

**Figure 2:**
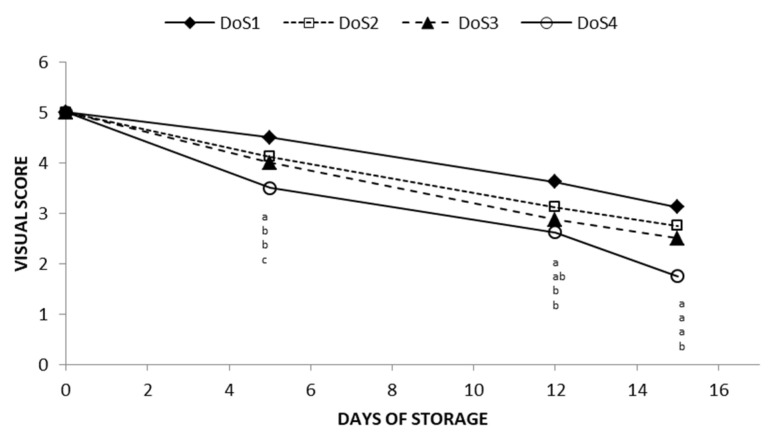
Visual score values of fresh-cut apples treated with four different DoS stored for 15 days at 5 °C. Different letters indicate statistical differences among the treatments on every sampling day as resulted by Tukey’s test.

Two-way ANOVA highlighted the significance of physical parameters for the different treatments proposed, the storage time and their interaction. For all of them, the mean value associated with the lowest force required for cutting operations, that is 30 N (DoS1), statistically differed from the value obtained with the remaining forces. In fact, 100 N, 140 N, and 190 N, which correspond, respectively, to DoS2, DoS3, and DoS4 treatments, did not show any statistical significance. In particular, the browning index, defined as the pureness of brown, pointed out a remarkable difference among the treatments. This result is in accordance with the augmentation of a* and b* values and the reduction of the L* value and Hue angle. The saturation values (Chroma) showed a significant effect for each factor considered. The visual appearances support the previously discussed parameters, indicating that apple slices cut with the sharpest knife (DoS1) maintain better overall quality compared to the other treatments, especially DoS4. Specifically, the RGB images and the score differences of the samples over the storage period are detailed in Figure 1 and Figure 2, respectively.

On day 5, the visual score for DoS1 showed a slight reduction from the initial value of 5, whereas, DoS2, DoS3, and DoS4 showed a significant decrease, with scores dropping to 4.1, 4, and 3.5, respectively, with DoS4 nearly reaching the limit of marketability (score 3) after the first sampling. After 12 days, only the samples treated with DoS1 remained marketable, while the scores for the other treatments fell below the marketability threshold, with DoS4 almost reaching the limit of edibility (score 2). By the end of the storage period, only the slices cut with the sharpest blade remained marketable, whereas the slices cut with blunter blades, particularly those with the DoS3 and DoS4, had mean scores of 2.5 and 1.75, respectively.

**Figure 3:**
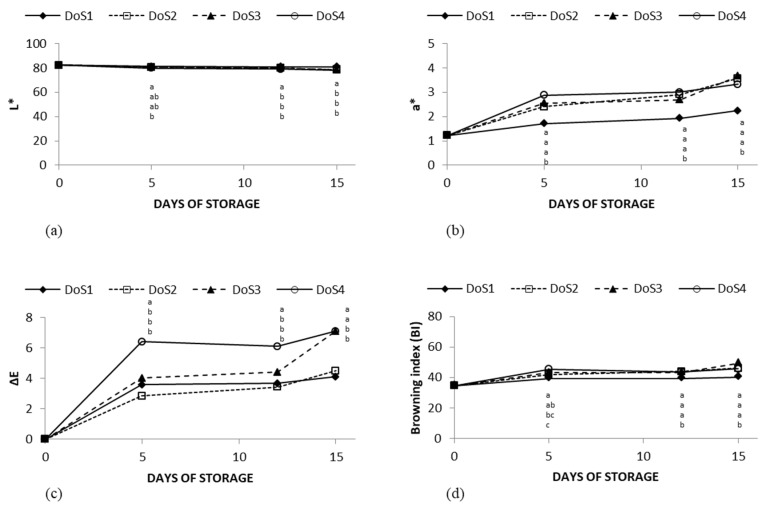
(**a**) L*, (**b**) a*, (**c**) ΔE, and (**d**) browning indices of fresh-cut “Golden Delicious” apples treated with four different DoS stored for 15 days at 5 °C. Different letters indicate statistical differences among the treatments on every sampling day as resulted by Tukey’s test.

The trend of the physical parameters L*, a*, and ΔE and the browning indices are shown in Figure 3, representing the effects of different cutting treatments.

Different DoS significantly affected the physical parameters of the fresh-cut apples. As pointed out in Table 1, the treatment performed with optimum sharpening conditions of the knife remarkably affected the physical parameters, as shown in the graph above where intermediate cutting forces resulted in values more similar to the one obtained with the dull knife. The apples cut with a sharper knife, represented by DoS1, showed a better appearance in terms of lightness with a decrease in the L* value of 1.6%, from 82.14 to 80.81 after 15 days of storage. On the other hand, the treatment performed with a blunt knife led to a significant decrease in the L* value, which was recorded to be 78.08 at the end of the storage time, decreasing from day 0 by 4.9% (Figure 3a). In accordance with the trend just discussed, the a* values did not change during the storage time for DoS1, unlike the DoS4 treatment where the values increased from 1.22 to a higher value of up to 3.32 (Figure 3b), indicating the increase of the red index. Hence, higher values of L* together with low values of a* portrayed a better quality of the product in terms of color. On the basis of L*, a*, and b* values, the ΔE index indicated an intense change of color after 15 days of storage when the fresh-cut apples were treated with the DoS4, while with the DoS1 the difference between day 0 and 15 was slightly appreciable (Figure 3c). Lastly, Figure 3d on the browning index resumes and confirms what has been explained so far. According to results obtained, the use of a sharp cutting blade preserved the visual quality of the products, with stable browning index values during the storage time; in contrast to the cut performed with a dull blade, where the index reached a value of 45.74 after starting at 34.76.

**Figure 4:**
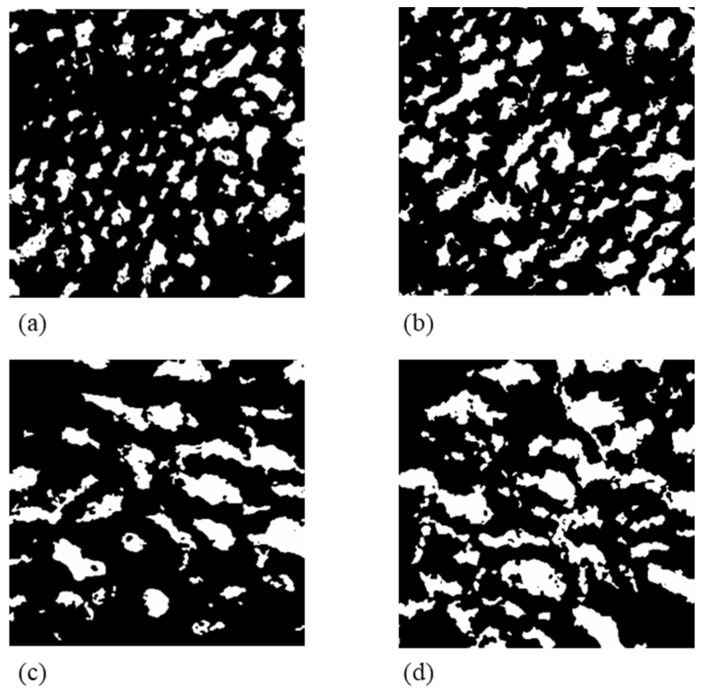
Binary 2D-X-ray images of cutting surface (**left**) and base (**right**) of “Golden Delicious” fresh-cut apples samples treated with DoS 1 (**a**,**b**) and DoS 4 (**c**,**d**) on day 0.

Similar results were obtained for other apples varieties [31,32], such as pitaya fruits [33], radishes [34], lemons [35], eggplants [10], and carrots [36], where the explanation of this phenomenon can be found in the chemical patterns activated after cutting. A wound, in fact, activates and increases phenylalanine ammonia-lyase (PAL) activity [29], triggers secondary metabolism, and promotes physiological and morphological responses such as membrane lipid degradation and water loss [37]. Moreover, when fresh-cut products are cut with sharper tools, fewer layers of the plant tissue are compromised, minimizing the effects of mechanical damage. To better study and to elucidate the potential damage generated during cutting with tools of different degrees of sharpness, 2D X-ray images of the microstructures of the apple tissues were elaborated on, as shown in Figure 4. Considering the white pixels and the black pixels, respectively, represent the void phase (pores) and the solid phase (cell membranes and native liquids), the overall comparison between the X-ray images reveals significant differences in the microstructure of apples after cutting with a blunt or edge blade.

The images show that by using a blunt blade (DoS4), the microstructure shows a reduced void phase at both the cutting surfaces of the sample. The pores of such samples appeared in a limited number but larger than in the piece cut with a sharp blade (DoS1). On the other hand, the solid fraction increased as well as the average dimensions of the solid elements of the apple tissues. Contrarily, for the DoS1 samples, a higher porosity fraction characterized by several small pores which break the solid phase into several phases and small structural elements of the apple tissues have been observed. Probably, the structural damage generated by using the blunt edge promoted the release of native liquids from the cytoplasm to the pores and, therefore, the creation of additional fractures, making the solid phase more complex and heterogeneous, characterized by more packed and fractured cells membranes. Contrarily, the samples treated by an edgy blade exhibited a different microstructure characterized by a high number of pores with a smaller dimension.

**Figure 5:**
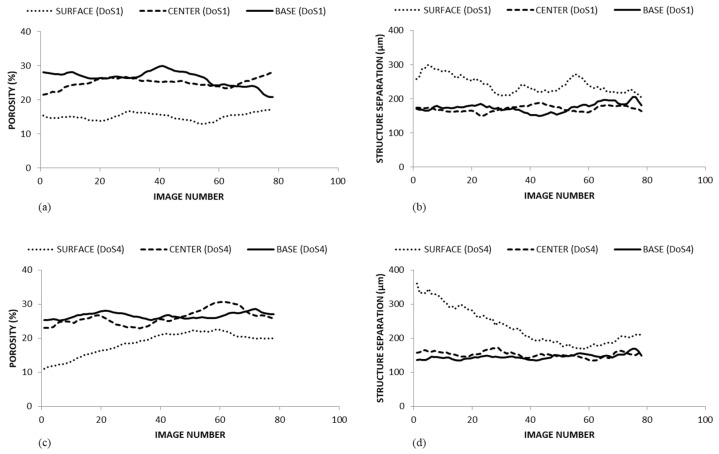
Porosity fraction (%) and Structure Separation (µm) on the surface, center, and base of “Golden Delicious” fresh-cut apples samples treated with DoS 1 (**a**,**b**) and DoS 4 (**c**,**d**) on day 0.

Our observations are in accordance with Ting et al. [38] and Mosqueda-Melgar and Tapia [39] who reported that solid cell-to-cell adhesion in fresh apples with small intercellular spaces are more prone to breakdown when subjected to wound stress, causing a disruption of the membranes, the loss of compartmentalization, and the leakage of cytoplasm into intercellular spaces. To better elucidate the changes in the microstructure properties of the apple tissues, Figure 5 shows the changes of the porosity fraction and the structure separation of the samples along the Z direction. More specifically, the figures show a total of 77 slices for three different portions of the apple cortex, literally the top, the center, and the bottom of the samples, corresponding to 0.5 mm of tissue.

At first, by comparing the samples, DoS1 shows higher porosity fractions ranging between 13% and 30%, while lower values were observed for the DoS4 sample ranging between 11% and 30%. However, considering the first layers of apple tissues—i.e., the first 80 images—the average lower porosity fraction values observed on the surface of the apple samples treated with DoS4 sustains the aforementioned hypothesis of greater structural damage.

The figure plots (Figure 5b,d) represent the structure separation (µm)—i.e., the average distance of the void elements in the apple slice—when the samples were treated with a sharp (DoS1) or blunt (DoS4) blade. According to the previous results, the average dimension of the solid elements of the samples was in the range of 135 and 360 µm for the sample treated with a blunt blade while the values were significantly lower for the DoS1 sample—in the range of 150 to 299 µm. More specifically, when a sharp blade was utilized the structure separation index on the surface of the samples showed a value in the narrow range of 210 to 299 µm, while a much greater variability between 360 and 195 µm was observed for the sample treated with a blunt blade.

The obtained results confirm the importance of the cutting procedure, suggesting that the use of a blunt cutting tool increases the damage to apples tissues, with a negative effect on the appearance while increasing the degradation reactions occurring during their shelf-life.

Table 2 shows the average values of the porosity fraction and structure separation index of several apple samples (n.3) on the first layers of apple tissues treated with the DoS1 and DoS4. The results show a clear distinction between the two treatments, even with a larger number of samples: the DoS1 exhibited an higher average porosity compared to the DoS4, while the structure separation index was significantly greater for the DoS4. These findings are in accordance with the previous discussion.

According to these findings, further experiments are needed to precisely explain the microstructural damages of fruits and vegetables as a result of cutting with different tools and knife materials, also considering the biological variations in the solid and void phases distribution within the fruit [26]. Herremans et al. [40], in their study on ‘Jonagold’ apples, showed that porosity, defined as the ratio of intercellular spaces to the total volume [38], increases gradually from the core to the peel of an apple. Furthermore, the void volume increases during growth; also, the void disposition changes from cultivar to cultivar, whereas cells become flabby with a reduction in the adhesions among them [38].

As per our knowledge, the correlation between degree of sharpness and micro-CT have not been investigated.

Ready-to-eat fruits have different return to wound stresses, depending on cultivar, maturity stage, and storage conditions, that promote biochemical and physiological alterations, increasing the ethylene content and respiration rate, as well as causing a firmness reduction and browning [41]. The phenomenon that underlays the physiological and structural disorders is the disruption of the middle lamella structure caused by the actions of pectinolytic and proteolytic enzymes which are activated with mechanical damage [39]. This systemic modification entails one of the main limiting factors in the fresh-cut apple process, that is browning, thanks to the direct contact between enzymes like polyphenol oxidase (PPO) and phenolic compounds assisted by O_2_ flow into the wound tissue [37]. It is conceivable to infer that a high number of cells are disrupted when a cutting procedure is carried out with a blunt cutting tool, inducing more efflux of fluids into hallow spaces and more severe physiological and morphological changes.

## 4. Conclusions

In the present study, it has been demonstrated that optimal results can be achieved when cutting operations on fresh-cut products are performed with sharp knives. The data underline how the use of a sharp cutting tool significantly reduces physical symptoms, particularly browning, supported by the results obtained from X-ray micro-CT. Ultimately, this could be a hopeful base for further research to better understand the threshold above which, both from a physical and microstructural point of view, wound stress becomes harmful for fresh-cut products.

## Figures and Tables

**Table 1:** Effect of treatment (A), time (B), and their interaction on quality attributes of “Golden Delicious” apples during storage. Within each row, each factor and their interaction have a significant effect for *p* ≤ 0.05 (*); *p* ≤ 0.01 (**); and *p* ≤ 0.0001 (****); otherwise, it is not significant (ns). In each row different letters indicate statistical differences as resulted by Tukey’s test.

	30 N	100 N	140 N	190 N	A: Treatment	B: Time	A × B
L*	81.5 a	79.9 b	80.1 b	79.9 b	****	****	****
a*	1.6 b	2.7 a	2.6 a	2.5 a	****	****	**
b*	25.4 b	26.7 a	27.0 a	26.5 a	****	****	*
Chroma	25.4 b	26.9 a	27.1 a	26.7 a	****	****	**
Hue angle	86.4 a	84.4 b	84.7 b	84.7 b	****	****	**
Browning index	38.1 b	42.5 a	42.8 a	42.0 a	****	****	**
Visual score	4.1 a	3.8 b	3.6 b	3.2 c	****	****	****

**Table 2:** Porosity fraction and structure separation index of 3 apples samples treated with a sharp (DoS1) or blunt (DoS4) blade. Data represent the microstructure properties of the first 0.5 mm of the sample from the cutting surface.

	DoS1	DoS4
Porosity	14.12 ± 1.5	11.43 ± 5.21
Structure separation index	280 ± 8.75	332.43 ± 25.34

## Data Availability

The original contributions presented in the study are included in the article, further inquiries can be directed to the corresponding author.

## References

[1] Ansah F.A., Amodio M.L., De Chiara M.L.V., Colelli G. (2018). Effects of equipment and processing conditions on quality of fresh-cut produce. J. Agric. Eng..

[2] Francis G., Gallone A., Nychas G.J., Sofos J.N., Colelli G., Amodio M.L., Spano G. (2012). Factors affecting quality and safety of fresh-cut produce. Crit. Rev. Food Sci. Nutr..

[3] Incardona A., Fatchurrahman D., Amodio M.L., Colelli G. (2022). Reducing Mechanical Damage Induced by Fresh-Cut Processing. Italus Hortus.

[4] Jacobo-Velázquez D.A., Martínez-Hernández G.B., Del S., Rodríguez C., Cao C.-M., Cisneros-Zevallos L. (2011). Plants as Biofactories: Physiological Role of Reactive Oxygen Species on the Accumulation of Phenolic Antioxidants in Carrot Tissue under Wounding and Hyperoxia Stress. J. Agric. Food Chem..

[5] Caretto S., Linsalata V., Colella G., Mita G., Lattanzio V. (2015). Carbon fluxes between primary metabolism and phenolic pathway in plant tissues under stress. Int. J. Mol. Sci..

[6] Brecht J.K., Saltveit M.E., Talcott S.T., Schneider K.R., Felkey K., Bartz J.A. (2004). Fresh-cut vegetables and fruits. Horticultural Reviews.

[7] Singh V., Das M., Das S.K. (2016). Effects of knife edge angle and speed on peak force and specific energy when cutting vegetables of diverse texture. Int. J. Food Stud..

[8] Portela S.I., Cantwell M.I. (2001). Cutting blade sharpness affects appearance and other quality attributes of fresh-cut cantaloupe melon. J. Food Sci..

[9] Surjadinata B.B., Cisneros-Zevallos L. (2003). Modeling wound-induced respiration of fresh-cut carrots (*Daucus carota* L.). J. Food Sci..

[10] Mishra B.B., Gautam S., Sharma A. (2012). Browning of fresh-cut eggplant: Impact of cutting and storage. Postharvest Biol. Technol..

[11] Incardona A., Amodio M.L., Colelli G. (2021). Monitoring the effect of cutting blade sharpness on quality of fresh-cut product. Acta Hortic..

[12] McCarthy C., Hussey M. (2006). On the sharpness of straight edge blades in cutting soft solids: Part I—Indentation experiments. Eng. Fract. Mech..

[13] McCarthy C.T., Annaidh A.N., Gilchrist M.D. (2010). On the sharpness of straight edge blades in cutting soft solids: Part II—Analysis of blade geometry. Eng. Fract. Mech..

[14] Lim K.S., Barigou M. (2004). X-ray micro-computed tomography of cellular food products. Food Res. Int..

[15] Wang Z., Herremans E., Janssen S., Cantre D., Verboven P., Nicolaï B. (2018). Visualizing 3D food microstructure using tomographic methods: Advantages and disadvantages. Annu. Rev. Food Sci. Technol..

[16] Olakanmi S., Karunakaran C., Jayas D. (2023). Applications of X-ray micro-computed tomography and small-angle X-ray scattering techniques in food systems: A concise review. J. Food Eng..

[17] Du Z., Hu Y., Ali Buttar N., Mahmood A. (2019). X-ray computed tomography for quality inspection of agricultural products: A review. Food Sci. Nutr..

[18] Prawiranto K., Defraeye T., Derome D., Bühlmann A., Hartmann S., Verboven P., Nicolai B., Carmeliet J. (2019). Impact of drying methods on the changes of fruit microstructure unveiled by X-ray micro-computed tomography. RSC Adv..

[19] Erkinbaev C., Ramachandran R.P., Cenkowski S., Paliwal J. (2018). A comparative study on the effect of superheated steam and hot air drying on microstructure of distillers’ spent grain pellets using X-ray micro-computed tomography. J. Food Eng..

[20] Contardo I., Bouchon P. (2018). Enhancing Micro-CT methods to quantify oil content and porosity in starch-gluten matrices. J. Food Eng..

[21] Karmoker P., Obatake W., Tanaka F., Tanaka F. (2019). Visualization of porosity and thermal conductivity distributions of Japanese apricot and pear during storage using X-ray computed tomography. Eng. Agric. Environ. Food.

[22] Nugraha B., Verboven P., Janssen S., Wang Z., Nicolaï B.M. (2019). Non-destructive porosity mapping of fruit and vegetables using X-ray CT. Postharvest Biol. Technol..

[23] Chen Y., Parrilli A., Jaedig F., Fuhrmann A., Staedeli C., Fischer P., Windhab E.J. (2021). Micro-computed tomography study on bread dehydration and structural changes during ambient storage. J. Food Eng..

[24] Nugraha B., Verboven P., Janssen S., Hertog M.L., Boone M., Josipovic I., Nicolaï B.M. (2021). Oxygen diffusivity mapping of fruit and vegetables based on X-ray CT. J. Food Eng..

[25] Janssen S., Verboven P., Nugraha B., Wang Z., Boone M., Josipovic I., Nicolaï B.M. (2020). 3D pore structure analysis of intact ‘Braeburn’ apples using X-ray micro-CT. Postharvest Biol. Technol..

[26] Chigwaya K., Karuppanapandian T., Schoeman L., Viljoen D.W., Crouch I.J., Nugraha B., Verboven P., Nicolaï B.M., Crouch E.M. (2021). X-ray CT and porosity mapping to determine the effect of ‘Fuji’ apple morphological and microstructural properties on the incidence of CO_2_-induced internal browning. Postharvest Biol. Technol..

[27] Vicent V., Verboven P., Ndoye F.T., Alvarez G., Nicolaï B. (2017). A new method developed to characterize the 3D microstructure of frozen apple using X-ray micro-CT. J. Food Eng..

[28] Cefola M., Amodio M.L., Cornacchia R., Rinaldi R., Vanadia S., Colelli G. (2010). Effect of atmosphere composition on the quality of ready-to-use broccoli raab (*Brassica rapa* L.). J. Sci. Food Agric..

[29] Amodio M.L., Derossi A., Colelli G. (2015). Modeling phenolic content during storage of cut fruit and vegetables: A consecutive reaction mechanism. J. Food Eng..

[30] https://www.microphotonics.com/wp-content/uploads/2016/01/CTAn_parameters.pdf.

[31] Yildiz G., Palma S., Feng H. (2019). Ultrasonic cutting as a new method to produce fresh-cut red delicious and golden delicious apples. J. Food Sci..

[32] Chung H.S., Jeong M.C., Moon K.D. (2012). Effects of cutting methods on qualities of fresh-cut apples and leafy vegetables. Korean J. Food Preserv..

[33] Li X., Long Q., Gao F., Han C., Jin P., Zheng Y. (2016). Effect of cutting styles on quality and antioxidant activity in fresh-cut pitaya fruit. Postharvest Biol. Technol..

[34] del Aguila J.S., Sasaki F.F., Heiffig L.S., Ortega E.M.M., Jacomino A.P., Kluge R.A. (2006). Fresh-Cut Radish Using Different Cut Types and Storage Temperatures. Postharvest Biol. Technol..

[35] Artés-Hernández F., Rivera-Cabrera F., Kader A.A. (2007). Quality Retention and Potential Shelf-Life of Fresh-Cut Lemons as Affected by Cut Type and Temperature. Postharvest Biol. Technol..

[36] Du W.-X., Avena-Bustillos R.J., Breksa III A.P., McHugh T.H. (2012). Effect of UV-B Light and Different Cutting Styles on Antioxidant Enhancement of Commercial Fresh-Cut Carrot Products. Food Chem..

[37] Chung H.-S., Moon K.-D. (2009). Browning Characteristics of Fresh-Cut ‘Tsugaru’ Apples as Affected by Pre-Slicing Storage Atmospheres. Food Chem..

[38] Ting V.J.L., Silcock P., Bremer P.J., Biasioli F. (2013). X-Ray Micro-Computer Tomographic Method to Visualize the Microstructure of Different Apple Cultivars. J. Food Sci..

[39] Mosqueda-Melgar J., Tapia M.S. (2010). Edible Coatings as Carriers of Food Additives on Fresh-Cut Fruits and Vegetables. Stewart Postharvest Rev..

[40] Herremans E., Verboven P., Hertog M.L.A.T.M., Cantre D., van Dael M., de Schryver T., Nicolaï B.M. (2015). Spatial Development of Transport Structures in Apple (*Malus × domestica* Borkh.) Fruit. Front. Plant Sci..

[41] Soliva-Fortuny R.C., Grigelmo-Miguel N., Hernando I., Lluch M.A., Martín-Belloso O. (2002). Effect of Minimal Processing on the Textural and Structural Properties of Fresh-Cut Pears. J. Sci. Food Agric..

